# Understanding frontline employees’ fuzzy request behavior based on image theory

**DOI:** 10.3389/fpsyg.2025.1672141

**Published:** 2026-01-14

**Authors:** Xiaodong Li, Jingwen Duan, Qi Li, Ai Ren

**Affiliations:** 1 School of Economics and Management, Anhui Polytechnic University, Wuhu, China; 2 School of Business, State University of New York at New Paltz, New Paltz, NY, United States

**Keywords:** frontline employees, fuzzy request behavior, image theory, strategic image, trajectory image, value image

## Abstract

**Introduction:**

While frontline employees (FLEs) often make fuzzy requests (made during the service process and are outside of service policy or FLEs’ job descriptions or are not addressed by service providers, but are not unacceptable), there is no research explaining how such requests emerge. This research pioneers in using image theory to elucidate the underlying dynamics that propel the mechanism behind FLEs’ fuzzy request behavior.

**Methodology:**

The hypotheses were examined by using data from 324 FLEs who asked customers to share tables in restaurants.

**Results:**

The results show that normative legitimacy, role confidence, expected responsiveness, and expected technical quality have significantly positive impacts on request behavior, whereas service rule commitment has a significantly negative impact. Meanwhile, trajectory image (represented by expected responsiveness and expected technical quality) mediates the relationship between value image (represented by normative legitimacy and role confidence) and request behavior. Additionally, habit moderates the relationship between expected responsiveness and request behavior while service rule commitment moderates the relationship between expected technical quality and request behavior.

**Discussion:**

This study is the first to demonstrate the mechanism of FLEs’ fuzzy requests’ emergence and provides vital managerial implications to manage request behavior.

## 1 Introduction

Frontline employees (FLEs), customers, and service systems are the key elements in service encounters (Sivakumar et al., 2014). The marketing literature has long recognized the key roles of FLEs and their critical impacts when providing services (Wang et al., 2012). One such impact arises from the fact that FLEs may have divergent interpretations of the service delivery; consequently, FLEs may try to take advantage of the service system or customers to increase service capacity and efficiency or may just follow their own style in delivering service (Di Mascio, 2010; Li et al., 2018; Ng et al., 2016). One result of divergent interpretations is the emergence of fuzzy requests (hereafter, “requests” at some instances), which are common in our everyday encounters. For instance, during a dining service, if the items requested by the customer are out of stock, the FLEs will send them a request to specify replacement items. Another example is that when takeaway staff deliver food, in most cases the delivery is scheduled according to the worker’s schedule rather than the customer’s preferred time.

The occurrence of fuzzy requests from FLEs and the possible issues these requests may cause have been investigated in previous studies. For example, FLEs’ fuzzy requests generally involve intentionally taking advantage of a customer (e.g., sharing service rooms, immediate cooperation with the employee, or asking for positive feedback), but it is an innocent problem for customers (Li et al., 2018; Wang et al., 2012). That is, FLEs adopt fuzzy requests in order to deliver adequate service rather than to delight customers (Hsieh et al., 2013; Zeithaml et al., 1993). The subjects that have captured research attention so far have primarily focused on customers’ response to fuzzy requests (Li et al., 2018; Teng et al., 2020) or on service performance improvement impact of these requests (Li et al., 2022). However, a lack of understanding of why FLEs send fuzzy requests limits the management of these requests. In practice, while appropriate fuzzy requests can benefit FLEs and service providers, inappropriate requests may lead to negative responses (e.g., refusal) or even service failure (Kostopoulos et al., 2014; Li et al., 2018; Söderlund and Mattsson, 2015). However, distinguishing between “appropriate” and “inappropriate” fuzzy requests in practice is itself a complex challenge. In addition, FLEs often work in service environments marked by uncertainty, time pressure, and incomplete information. In such settings, fuzzy request behavior—defined as flexible, non-scripted requests made by employees to customers or colleagues—has become an increasingly common service strategy. If service providers want to effectively guide and regulate this behavior, a fundamental first step is to understand which factors systematically influence employees’ willingness and tendency to make such requests. In other words, clarifying the antecedents of fuzzy request behavior is the theoretical foundation for developing management strategies and preventing service failures caused by inappropriate requests. At present, these key antecedents remain unclear. Therefore, this study aims to explain the mechanism by constructing and testing a comprehensive factorial model that identifies the factors influencing FLEs’ fuzzy request behavior. This provides the necessary theoretical basis for further exploration of the boundaries of appropriateness and related management interventions. Thus, it is critical to understand the mechanism underlying FLEs’ fuzzy request behavior (hereafter, “request behavior”).

Understanding these antecedents is both theoretically and practically important because inappropriate fuzzy requests do not occur randomly; rather, they arise from systematic cognitive and contextual processes, such as misjudging normative legitimacy, having inaccurate role confidence, or holding distorted expectations about service outcomes. Without clarifying these underlying mechanisms, organizations lack the diagnostic tools needed to prevent dysfunctional behaviors before they happen. Therefore, this study examines the cognitive and motivational foundations of FLEs’ fuzzy request behavior, aiming to explain not only when such behaviors occur but also why they are constructive in some contexts and dysfunctional in others.

Drawing from the image theory, we proposed a model to elucidate request behavior and its driving variables. Focusing on instances wherein FLEs asked customers to share tables in restaurants, request behavior data were collected by investigating 324 FLEs. We empirically examined the effects of normative legitimacy, role confidence, expected responsiveness, expected technical quality, habit, and service rule commitment on request behavior, and also analyzed the mediating and moderating effects among these antecedents.

This study makes several contributions by identifying the value-based, goal-based, and strategic cognitive drivers underlying request behavior. It is one of the first that focuses on requests from FLEs’ perspective and is the first to illustrate the driving mechanism for FLEs’ request behavior, which has not been considered in prior work. Additionally, with the assistance of image theory, our study provides a well-considered model for explaining how we can understand such requests from FLEs and what influences such request behavior. It provides a theoretical explanation for the complex dynamics that drive request behavior in service fluctuation settings. Lastly, our work provides practical insights for organizations in recognizing and managing the phenomenon of FLEs’ fuzzy request (behavior), as well as for FLEs themselves in making decisions regarding such request behaviors.

## 2 Literature review

### 2.1 Fuzzy requests from FLEs

The term “fuzzy requests” was first articulated by Wang et al. (2012) and it pertains to requests made during the service process that are slightly outside of service policy or FLEs’ job descriptions or are not addressed by service providers, but which are not unacceptable. Teng et al. (2020) extended the study of these requests and defined them as tactics or techniques that deviate slightly from the typical range of services but are not illicit, and which employees adopt to increase work performance or for other purposes. In contrast to the tight contents of a service script and unlike mistreatment or fraudulence, in which FLEs are completely in the wrong, employees generally understand the rules of conduct as flexible (Victorino and Bolinger, 2012). In this study, drawing on the research of Li et al. (2018) and Teng et al. (2020), we define FLEs’ fuzzy requests as requests made by FLEs, which slightly deviate from the service script or rules but are not acceptable to customers in service encounters. Generally, FLEs’ requests aim to enhance service efficiency, increase service capacity, or serve as a tool to ultimately improve the overall customer experience. Accordingly, the behavior of FLEs making these requests is referred to as fuzzy request behavior (i.e., “request behavior”).

Some limited research has enhanced our understanding of employees’ request behavior. For example, in studying the context of express pickup, Li et al. (2018) argued that delivery people do not obey the assigned delivery time or place specified online but ask customers to pick up their goods in the way the delivery person recommends; the researchers also consider such requests as situational and spontaneous in some cases. Teng et al. (2020) see these fuzzy requests as FLEs’ solutions to certain situations or as strategies to increase service capacity. Jones et al. (2014) proposed that due to the pressure of the rewards and punishments associated with customers’ positive evaluations and satisfaction with the provided service, FLEs sometimes request customers to give a positive evaluation.

Service interactions are dynamic and diverse (Li et al., 2022), which needs employees to be flexible in handling unexpected situations or special customer requests (Memon et al., 2025). The FLEs can exhibit positive behaviors to enhance customer experience or behave negatively to take advantage of customers or service rules. Some related literature can assist us in recognizing the driving forces of FLEs’ request behavior. For example, Leischnig and Kasper-Brauer (2015) found that employees’ personal attributes and work perceptions contribute predicting service-offering adaptive behavior in service encounters, while FLEs’ adaptive behavior coping with some special requests improves customers’ loyalty (Chen, 2023). Hoang et al. (2023) identified that intrinsic motivation has a direct influence on FLEs’ innovation behavior. Meanwhile, FLEs’ unethical behavior, which can be the bottom level of request behavior, can be driven by personal, organizational, and contextual factors (Jha and Singh, 2023). For example, behavioral norms, psychological safety, and working atmosphere are identified as the determinants of employees’ unethical behavior (Gupta et al., 2024). Additionally, some studies found that deviant behavior, which is similar to request behavior in violating rules, is influenced by leadership style (Valle et al., 2019), organizational culture (Rizani et al., 2022), and environment (Khattak et al., 2021; Zulfiqar et al., 2023).

While previous studies have enhanced the understanding of FLEs’ behaviors in service encounters, there are still some limitations. First, the existing studies mostly focused on the outcome of employees’ behavior, or gave some explanations for request behavior, which mainly focused on customers’ responses to the requests or relative service outcome, and there was no comprehensive and clear understanding of how such requests emerge from the FLEs. Second, some results on FLEs’ positive or negative behavior cannot be directly used for elaborating request behavior, the service outcome of which is uncertain (i.e., service delight, complement, or failure). Considering that request behavior is often context-driven or the response of a sudden service interaction (Teng et al., 2020), it is necessary to focus on FLEs’ perception of value image toward service encounters (Wang et al., 2012). Thus, we intend to propose a model to elucidate the mechanism of FLEs’ request behavior occurrence based on the image theory.

### 2.2 Image theory

Image theory, proposed by Beach (1993a), is considered a critical supplement for traditional rational decision theory (Morrell, 2004). The best engagement with image theory lies in understanding a given decision through three images, namely the value image, trajectory image, and the strategic image, which reveal actions taken toward values and ideals rather than desired ends (Lipshitz, 1993). This theory assumes that the decisions made by an individual, whose analysis is grounded in the interaction dynamics and contextual content of a specific situation, can be either maintained or modified (Beach, 1993b; Beach and Mitchell, 1987).

We adopted image theory to elucidate the mechanism of employees’ request behavior for two reasons. First, in service interactions, employees make requests to demonstrate their value principles and ideal work goals, which facilitates better service decisions and, in turn, improves customer satisfaction. A situation in which FLEs make fuzzy requests is consistent with the situations where image theory has been productively used. This is mainly because these requests emerge while providing the service and are results of specific service context and service principles of FLEs. The decision to make a request is contingent upon the specific context and may change along with the context. There are also no strict criteria for making a request or not, but the drivers of the decision vary from one decision maker and one situation to another (Beach, 1993b). Second, image theory has been adopted to explain employees’ decisions and behavior in service scenarios. Pesta et al. (2005) used three experiments to examine the decision-making processes underlying performance evaluation, revealing that performance evaluation correlated directly with both favorable and unfavorable data. Nonetheless, in the case of promotion choices, a preliminary filtering phase was evident, where the selectors concentrated solely on adverse information (Pesta et al., 2005). Building on the image theory, Maier et al. (2024) delineated the process by which shocks lead users to cease a service by formulating a model of discontinuation, which serves as a tool for comprehending the dynamics of user behavior in diverse settings. Clark et al. (2002) used image theory (a non-normative model of decision making) to explain the behavior of sports fans. In summary, image theory has been adopted to explore individual behavior, which we can use to study the mechanism of FLEs’ request behavior emergence.

### 2.3 Conceptual model

Image theory proposes that the choice to engage in a behavior is primarily made based on a compatibility test that draws on the decision maker’s three images (Beach, 1993b). These three images are the fundamental principles for evaluating behavior options or candidates, and a potential choice can be tested against these images (Nelson, 2004). Thus, images are seen as decision makers’ cognitive structures, which guide their behavior (Lipshitz, 1993). With the assistance of image theory, our study identifies the drivers of FLEs’ request behavior in service encounters through contextualization.

#### 2.3.1 Value image

Value image is made up of an individual’s principles, which are associated with notions about the rightness or wrongness and the ideals of candidate behavior (Beach, 1993b; Lipshitz, 1993). In a service encounter, we use normative legitimacy and role confidence to reflect the notions of value image. More specifically, normative legitimacy refers to whether a request behavior conforms to social values and norms (Maier et al., 2024; Wang et al., 2012). It represents notions about request behavior’s acceptability through judgments about whether it is right or wrong. Role confidence refers to FLEs’ perception that they are doing a good job in delivering the service. It reflects employees’ ideal of service style and lays the foundations for a service goal, plan, or a new behavior. Considering that value image serves to internally generate possible plans and guides externally generated candidate behavior, we propose that normative legitimacy and role confidence would influence FLEs’ request behavior.

#### 2.3.2 Trajectory image

Trajectory image consists of “concrete goals that an individual attempts to achieve” (Lipshitz, 1993). It represents what a decision maker hopes to achieve for themselves personally or for the organization in a specific context. In service interactions, FLEs generally seek to provide good service quality in a given service context (Grönroos, 1984). Considering the importance of service efficiency and service results (Li et al., 2018), this paper uses expected responsiveness and expected technical quality for reflecting the goals associated with trajectory image.

More specifically, expected responsiveness refers to FLEs’ expectation that they will be part of the customer support and serve them timely. From the customers’ perspective, responsiveness indicates whether employees will help them and deliver prompt service (Zeithaml et al., 1988). From the employees’ perspective, responsiveness shows they want to deliver quick, immediate, and appropriate service (Chiang and Trimi, 2020). Therefore, expected responsiveness expresses the desired goal of FLEs. Similarly, expected technical quality is also a fundamental goal of service delivery (Li et al., 2018), as it assumes service completion. Moving into the context of fuzzy requests, through the goals associated with trajectory image, expected responsiveness and expected technical quality should influence request behavior.

#### 2.3.3 Strategic image

The strategic image is made up of various plans designed to guide the actor in reaching the objectives on the trajectory image (Beach, 1993b). In the context of requests, we adopted only one aspect of plans, that is, tactics. Tactics are used to implement or progress one’s goals, which can be reflected by habit and service rule commitment. Habit is developed through FLEs’ past service experience and is a synthesis of how they handled situations in the past (Venkatesh et al., 2012). Rules can also influence FLEs’ turnover behavior in critical service contexts (Nguyen et al., 2023). Service rule commitment denotes the level at which the FLEs are bound by service regulations regarding the way they treat customers (Wang et al., 2011). It indicates what manners employees actually use to deliver service compared to the service script. Different employees can use or explain similar service scripts through various manners. In addition, an employee’s use and interpretation choices can be tactics they use to advance their goals. Therefore, habit and service rule commitment as strategic image serve to guide FLEs’ decisions and may influence their request behavior.

In summary, we conceptualize fuzzy request behavior as the outcome of an evaluative process that integrates value images (what is appropriate), trajectory images (what outcomes are expected), and strategic images (how actions are habitually or normatively regulated). First, the value image reflects the fundamental principles, moral values, and ideals of decision-makers (Beach, 1993b) and acts as the standard for judging whether a behavior is “right or wrong.” Normative legitimacy captures the extent to which a request aligns with recognized societal and organizational values, directly reflecting the “correctness” dimension of the value image. Role confidence, or employees’ belief in their ability to fulfill their service role, forms the internal ideal that shapes their service principles and moral judgments. Together, these constructs represent the core components of the value image. Second, the trajectory image consists of the concrete goals that decision-makers seek to achieve in a given situation (Lipshitz, 1993). In service interactions, the two key goals for employees are maintaining service efficiency and ensuring high service quality. Expected responsiveness corresponds to the process-oriented goal of providing timely, non-delayed service. Expected technical quality corresponds to the outcome-oriented goal of delivering services that are professional and effective. These constructs capture the essential goal-focused elements of the trajectory image. Third, the strategic image reflects the plans and tactics used to achieve trajectory goals (Beach, 1993b). Habit represents the automatic behavioral routines formed through past successful experiences (Venkatesh et al., 2012), serving as an experiential tactic that guides behavior. Service rule commitment reflects the extent to which employees internalize and comply with organizational service rules, guiding them on “how they should act.” Together, these factors form the bridge between goals and actions.

Based on the discussion, the specific variables used in this study—normative legitimacy and role confidence for the value image; expected responsiveness and expected technical quality for the trajectory image; and habit and service rule commitment for the strategic image—are grounded in contextualized operationalization. We selected constructs that not only reflect the core theoretical essence of each image in image theory (Beach, 1993b), but are also especially relevant and measurable in the context of frontline service employees making or handing fuzzy requests customer requests. This approach ensures a strong alignment between image theory and the empirical phenomena examined in this study.

#### 2.3.4 Relationships among images

The tests of value, trajectory, and strategic images drive a decision that answers the questions “why,” “what,” and “how” (Lipshitz, 1993; Nelson, 2004). Meanwhile, Nelson (2004) indicated that there exists a linear relationship from value image to trajectory image, and to strategic image. Thompson and Dahling (2010) further show that potential influence relationships exist among the three images. Building on this insight, our study extends this line of research by examining the mediating and moderating processes that underlie—and may complicate—these linear relationships, drawing from the work of Nelson (2004) and Thompson and Dahling (2010). The details are presented below.

Usually, value image reflects a strategist’s ethics, morals, and basics. Targets are symbolized by the trajectory image since it captures the envisioned future and the ultimate goal of one’s pursuits (Beach and Mitchell, 1987; Gerbec, 2012). In the service delivery process, the value image can guide individuals’ behavior. Moreover, the goals that FLEs’ services seek to achieve are influenced by their personal principles and norms. The trajectory image translates the abstract principles in the value image into concrete, achievable goals, which are then translated into concrete behavior. Consistent with this point, we propose that trajectory image can mediate value image when they influence request behavior.

Additionally, trajectory image represents the aims of the decision maker, and the corresponding strategic image comprises the intended plan for attaining goal achievement (Beach and Mitchell, 1987; Maier et al., 2024). Furthermore, in contrast to Nelson’s (2004) study—which focuses on a derivative relationship among the three images (value, trajectory, and strategic), where principles give rise to goals and goals give rise to plans—this represents a static and constitutive logic. In this study, however, we further examine how a given plan (strategic image) can strengthen or weaken the influence of a given goal (trajectory image) on final behavior, which reflects a dynamic and effectual form of influence. These two forms of logic can theoretically coexist and are not mutually exclusive.

**Figure 1:**
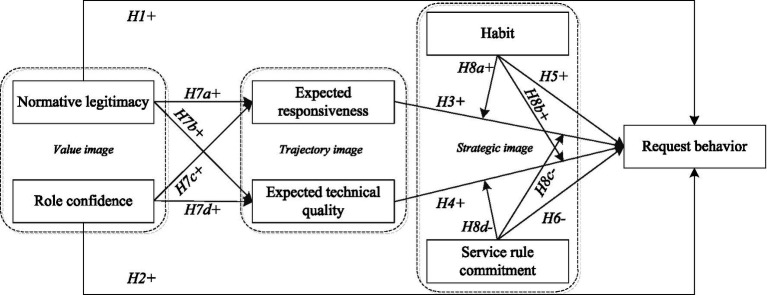
Conceptual model.

Moreover, Beach (1993b) notes that the quality of the strategic image is directly related to the efficiency with which the goals in the trajectory image are achieved. Specifically, in service interactions, employees’ perceptions of the service goals they expect to achieve for customers influence a range of behaviors throughout the service process. The strategic image helps decision makers determine what actions are necessary and how to effectively deploy resources and time to achieve them by providing a course of action, thus influencing decision makers to move from goals in the trajectory picture to specific behaviors. Trajectory images rely on strategic images. We also argue that strategic image can moderate the effects of trajectory image and request behavior (Beach, 1993b). Figure 1 shows the proposed conceptual model.

## 3 Hypotheses

### 3.1 Normative legitimacy

Normative legitimacy refers to whether a request behavior conforms to social values and norms (Wang et al., 2012). Individuals make a legitimacy test to assess whether their potential behavior fits the norms and values of a specific context, group, or organization (Ajzen and Driver, 1991). Drawing on social identity theory (Tajfel and Turner, 2004), individuals are motivated to maintain a positive self-concept and a sense of belonging by aligning their actions with the norms of their salient social groups, such as their organization or profession. When FLEs perceive a fuzzy request (e.g., asking customers to share tables) as normatively legitimate, they experience a sense of identity–organization congruence, in which their personal evaluation of the behavior aligns with the perceived values of their professional role and organizational environment. This congruence reduces internal role conflict and lessens the perceived social risk associated with deviating from standard service scripts.

Within the broader framework of image theory (Beach, 1993a, 1993b), normative legitimacy functions as a key component of the value image. The value image represents an individual’s principles and acts as a screening mechanism for evaluating potential behaviors. Prior studies have made some relevant exploration. For example, Wang et al. (2012) reported that employees’ compliance behavior is based on normative legitimacy of fuzzy requests. Customers’ responses to these requests also rely on employees’ reasonableness (Li et al., 2018). Therefore, FLEs assess the potential responses of customers, organizations, and even themselves and see whether their behavior fits normative legitimacy. Considering the uncertainness and unclearness of fuzzy requests, FLEs assess the normative legitimacy of a specific request to avert possible conflicts or signs of service breakdown, and the ability to avoid these undesired consequences serves as a fundamental condition for deciding whether to engage in a request behavior. Consequently, we suggest the following hypothesis:

*H1*: Normative legitimacy has a positive association with request behavior.

### 3.2 Role confidence

Role self-efficacy refers to individuals’ belief in their ability to perform a specific task (Söderhjelm et al., 2018). Accordingly, role confidence can be considered individuals’ understanding and perception of their role in the organization, which can be described as the confidence in their ability and efficiency to complete the tasks expected of the role. Self-efficacy theory (Bandura and Adams, 1977) suggests that confident FLEs perceive lower potential costs of failure and are therefore more willing to engage in risky or script-deviant requests. Higher self-efficacy increases employees’ willingness to try unconventional strategies. At the same time, high confidence functions as a “personal resource” that helps buffer against the psychological depletion that may result from unsuccessful requests, thereby making request behavior more likely.

From the perspective of image theory (Beach, 1993a, 1993b), role confidence is a key component of the value image. It reflects the principle that “I am competent and can manage risks in my role.” Fuzzy requests can be seen as risky tactics for pursuing service goals, as they can lead to service failures (Kostopoulos et al., 2014). However, Mittal et al. (2002) suggested that increased confidence among employees tends to result in riskier decisions and resource commitments, favoring negatively framed circumstances over positively framed ones. Thus, employees with role confidence would engage in positive behaviors to address service interactions. They have confidence that they can deal with customers’ problems and positively adapt their behavior accordingly or ask customers to adapt to the specific service situation (Geum et al., 2016). Along similar lines, Siemsen et al. (2009) revealed that confidence positively impacts workers’ knowledge-sharing behavior. Therefore, role confidence, grounded in self-efficacy, empowers FLEs by reducing the perceived risks associated with fuzzy requests and aligning such behavior with their professional self-concept, thereby increasing the likelihood that they will engage in it. Hence, we hypothesize the following:

*H2*: Role confidence positively related to request behavior.

### 3.3 Expected responsiveness

Expected responsiveness reflects the employee’s perspective on the timely response to customer needs and the prompt delivery of services (Chiang and Trimi, 2020). Expected responsiveness represents a concrete, high-priority goal within the service encounter. Drawing on goal-setting theory (Locke and Latham, 2015), FLEs use request behavior as a means to reduce customer wait time and provide immediate assistance. Goal-setting theory suggests that specific and challenging goals strongly motivate behavior by directing attention and effort toward activities that support goal attainment.

Furthermore, expectation-confirmation theory (Oliver, 1980) provides insight into the cognitive reinforcement mechanism underlying this process. When FLEs hold high expectations for responsiveness, they anticipate that their request will prompt immediate customer cooperation, thereby confirming the effectiveness of their action and quickly closing the service loop. This anticipated positive confirmation operates as a mental simulation of success, reinforcing the decision to act.

Within the overarching framework of image theory (Beach, 1993a, 1993b), expected responsiveness is a core element of the trajectory image, which specifies what the employee aims to achieve—in this case, a swift and smooth service process. During a context-driven service phenomenon, employees seek help from customers or make specific requests to adapt (Li et al., 2018). In settings where service providers exhibit an increased level of responsiveness, it is common that employees and customers adapt to each other. Through requests, employees can provide timely service feedback and even improve their care and help for customers by relying on their own and customers’ changing behaviors. Thus, we have the following hypothesis:

*H3*: Expected responsiveness is positively related to request behavior.

### 3.4 Expected technical quality

Expected technical quality refers to an individual’s anticipation or belief regarding the level of technical competence, accuracy, and effectiveness that another party will demonstrate in completing a specific task or delivering a service. Expected technical quality has been identified as the critical factor for service appraisal (Grönroos, 1984). Both customers and employees share a common goal of completing the service (Dagger et al., 2007). Drawing on service quality theory (Grönroos, 1984), we propose that employees are motivated to perform behaviors they believe will lead to high-quality outcomes. Expected technical quality reflects employees’ expectations regarding the successful completion and technical accuracy of service delivery. Therefore, when FLEs believe specific behaviors will minimize service interruptions and maximize service outcomes, these expected technical quality goals motivate them to fulfill the request.

Within the framework of Image Theory (Beach, 1993a, 1993b), expected technical quality is a fundamental component of the trajectory image. It represents a concrete, outcome-oriented goal (such as the successful and flawless completion of the service). The theory posits that decision-makers assess potential behaviors against their trajectory image using the compatibility test. For example, if a request is seen as a viable tactic to minimize disruptions and ensure service completion, it is deemed consistent with the trajectory image and is therefore more likely to be enacted.

In other words, expected technical quality serves as an important target for employees’ action. Considering that fuzzy requests generally aid in the completion of service delivery, FLEs would tend to make such requests. In addition, there is evidence from the literature that customers tend to comply with these requests because of expected technical quality (Li et al., 2018). Hence, we propose the following hypothesis:

*H4*: Expected technical quality has a positive relationship with request behavior.

### 3.5 Habit

The concept of habit encapsulates how readily FLEs carry out the act of issuing fuzzy requests without conscious thought (Venkatesh et al., 2012). Drawing on automaticity theory (Bargh and Chartrand, 1999; Verplanken and Aarts, 1999), research suggests that repeated past behavior becomes automatic and guides future behavioral responses with minimal cognitive effort. In service contexts, these habitual behaviors shape how employees respond to familiar scenarios. Within the framework of Image Theory (Beach, 1993a, 1993b), habit functions as a key element of the strategic image—the repertoire of plans and tactics an individual uses to achieve goals. A well-established habit represents a pre-formed, highly accessible “plan” that is automatically activated. Consequently, when a service scenario presents a cue similar to past situations, the habitual response is triggered immediately.

It is argued that habit guides one’s action through their experience linked to specific tasks (Sun et al., 2017). Though requests may not be very common events, FLEs with habit would still tend to engage in request behavior. Preceding studies have similarly disclosed that habit is a significant predictor of a particular behavior (Phau et al., 2014). Thus, we hypothesize:

*H5*: Habit is positively related to request behavior.

### 3.6 Service rule commitment

Service rule commitment signifies the level at which employees are dedicated to adhering to the established service guidelines regarding the treatment of customers (Wang et al., 2011). Drawing on self-determination theory (Deci and Ryan, 1980), service rule commitment defines the role scripts of employees and suppresses their “autonomy.” High service rule commitment means that FLEs strictly confine themselves within the role script, and “request behavior” may be regarded as behavior beyond the scope of the role or script. FLEs who strongly internalize prescribed role expectations are more likely to engage in in-role behaviors and less likely to deviate from standard scripts. Service rule commitment represents the extent to which FLEs internalize and strictly adhere to organizational service scripts. Therefore, highly committed employees tend to strictly adhere to service rules rather than proactively requesting modifications.

Within the framework of Image Theory (Beach, 1993a, 1993b), this high level of service rule commitment becomes a paramount principle within the employee’s value image—specifically, the value of “being a rule-abiding, compliant employee.” Simultaneously, it shapes a strategic image, where the primary and often sole “plan” for action is strict adherence to the service script. A request that deliberately deviates from the script is fundamentally incompatible with the value of compliance and contradicts the established strategic plan of rule-following. Consequently, it is highly likely to be rejected. Past research has pointed out that employees’ ideas concerning how they deliver services and influence service processes and results are internalized (Baker and Kim, 2021; Demirbag et al., 2012). In other words, the higher an employee’s service rule commitment is, the more the employee is inclined to think less and follow the rules/scripts more strictly. Otherwise, employees will take advantage of the ambiguity of service instructions and decide on their own behaviors. Consequently, we hypothesize:

*H6*: Service rule commitment has a negative association with request behavior.

### 3.7 Mediating effects between images

According to Image Theory (Beach, 1993a, 1993b), employees’ behavioral decisions are based on their cognitions. When FLEs make fuzzy requests, this decision-making process is jointly influenced by their value image (beliefs about what is right and proper) and their trajectory image (expectations of behavioral outcomes). Drawing on Social Cognitive Theory (Locke, 1987), when FLEs perceive their fuzzy request as normatively legitimate (i.e., it aligns with their value image), they will more actively anticipate that this behavior will lead to positive service outcomes (i.e., forming a positive trajectory image). This positive anticipation, in turn, motivates them to demonstrate better performance in the service process. It is argued that the more reasonable FLEs believe their fuzzy request to be, the more likely they are to expect positive results.

Regarding responsiveness, the service process itself is an important aspect of delivery. When FLEs perceive their means (such as making fuzzy requests) as reasonable and acceptable, they feel more confident. Due to this adaptation by FLEs and the expected adaptation by customers, FLEs are better positioned to provide timely and considerate service. When FLEs feel that the request behavior is feasible, they will strive for better service quality, which subsequently enhances customer satisfaction and meets the objectives of the request. In summary, normative legitimacy, as a core value image component, cultivates positive outcome expectations (trajectory image) by providing the moral and cognitive confidence necessary to envision both an efficient service process and a successful expected service outcome. Considering these points, we hypothesize:

*H7a*: Normative legitimacy has a positive relationship with service responsiveness.

*H7b*: Normative legitimacy is positively related to expected technical quality.

Similarly, drawing on Self-Efficacy Theory (Bandura, 1997), role confidence represents FLEs’ belief in their own service capability. This serves as a key value image, based on personal or organizational norms. When FLEs possess high role confidence in making fuzzy requests, it strengthens their belief that they can successfully execute the desired service process and achieve the outcomes (the trajectory image). Previous research has revealed that employees’ confidence positively impacts customers’ perception of service quality (De Jong et al., 2006). Employees’ confidence beliefs increase self-efficacy and help them make better and more timely service decisions, which result in superior service quality and heightened customer satisfaction (Wang and Netemyer, 2002). When employees feel confident, they may be proactive in responding to emergencies in the service process, eventually leading to higher expectations of service goals. We can infer that when role confidence enhances the performance of service delivery, employees would then have a higher expectation of responsiveness and technical quality. In essence, role confidence, as a manifestation of self-efficacy within the value image, empowers FLEs to form more positive and assured expectations regarding both the process and the outcome of their service delivery. We thus hypothesize:

*H7c*: Role confidence is positively related to expected responsiveness.

*H7d*: Role confidence has a positive relationship with expected technical quality.

### 3.8 Moderation effects between images

Habit forms automatically from environmental cues and varies along with feedback from the outer environment (Sun et al., 2017). Within the framework of Image Theory (Beach, 1993a, 1993b), habit can be viewed as a type of automatically activated strategic image, derived from past experience. In our research context, drawing on habit formation theory (Gardner et al., 2024), strong habits enable FLEs to remain in a state of readiness, allowing them to initiate request behavior and respond promptly in a nearly automated manner. When the habit is strong, it enhances employees’ confidence in achieving high-responsiveness and high-quality goals, making it more likely for these goals (trajectory images) to translate into actual request behavior. Conversely, when service habit is weak, this relationship is weakened. Because a weak habit indicates an undeveloped plan, it leads employees to hesitate, requires more cognitive effort, and results in a lack of confidence in execution, even if employees have highly responsive goals. So, their expectations of request behavior linked to service quality naturally weakens. Thus, we hypothesize:

*H8a*: Habit positively moderates the relationships between expected responsiveness and request behavior.

*H8b*: Habit positively moderates the relationships between expected technical quality and request behavior.

Commitment represents a mental disposition that inclines an individual to make considerable contributions to the organization (Angle and Perry, 1981; Lim et al., 2017). Service rule commitment entails employees’ agreement with the organization’s service goals and their ability to consistently follow these standards in their daily tasks (Wang et al., 2013). Grounded in Consistency Theory (Festinger, 1957; Kiesler, 1971), high service rule commitment establishes a ‘normative boundary’ for employee behavior. Employees with strong commitment prioritize the alignment between their actions and formal service rules (Kiesler, 1971), which constrains their ability to engage in extra-role behaviors to a certain extent. Therefore, even if FLEs have high expectations for response speed or technical quality, they will not easily translate these expectations into actual service request behaviors. In other words, high service rule commitment weakens the positive relationship between expected outcomes and request behavior. In other words, employees who demonstrate strong service rule commitment are able to consistently maintain a qualified, good-natured, and persistent demeanor with customers and are committed to aligning their actions with these standards, even in challenging situations (Wang et al., 2011). In the service delivery process, service rule commitment keeps employees highly compliant. Employees who exhibit strong dedication to service rules follow service scripts to perform services and make decisions, and they are not concerned with too many requests or fuzzy requests, as such requests can lower employees’ expectations of responsiveness and technical quality. On the contrary, when the service rule commitment is lower, employees learn from these requests, which means they have a better chance of getting the desired results, and their expectations of service-related request behavior naturally decrease. Thus, we hypothesize:

*H8c*: Service rule commitment negatively moderates the relationships between expected responsiveness and request behavior.

*H8d*: Service rule commitment negatively moderates the relationships between expected technical quality and request behavior.

## 4 Methodology

### 4.1 Procedures

To verify the hypotheses, we chose a research context in which customers in chain restaurants in the entertainment market were asked to share tables, and we chose this context for three reasons. First, restaurant food service has representative and universal characteristics of the service industry (Li et al., 2021). Second, the way in which employees interact has a direct impact on the faithfulness of patrons, which could be pivotal in deciding the sustainability of the restaurants. Third, sharing tables is pervasive in restaurant service.

To prevent or decrease common method bias, data were collected twice by recording respondents’ WeChat IDs from March 17 to April 20 (for normative legitimacy, expected responsiveness, habit, and request behavior) and then from May 1 to May 18 (for role confidence, expected technical quality, and service rule commitment). Of the 500 questionnaires administered, 324 were complete and fit the initial check, achieving a response rate of 64.8%. Participants who successfully finished the survey on two occasions were rewarded with a 20 RMB supermarket voucher. A summary of the sample characteristics is given in Table 1. T-tests were employed to examine the variance in item responses between the last 50 cases to check for non-response bias (Li et al., 2018). The findings revealed no significant differences, suggesting that non-response bias is not a considerable problem within the study context.

**Table 1:** Sample characteristics.

Variable		Frequency^a^	Percentage (%)
Gender	Female	176	54.32
	Male	148	45.68
Age	16–25 years old	105	32.41
	26–35 years old	97	29.94
	36–45 years old	90	27.78
	Over 45 years old	32	9.88
Work years	Less than 1 year	28	8.64
	[1, 2] years	159	49.07
	[3, 5] years	104	32.10
	More than 5 years	33	10.19

^a^*N* = 324.

### 4.2 Measures

Previous studies provided the base measures for the constructs in this study. Normative legitimacy was assessed using three measures taken from Wang et al. (2012). Role confidence was evaluated based on five items adapted from Yamamoto-Mitani et al. (2003). Four items for expected responsiveness were taken from Sun et al. (2012). Three items for expected technical quality were modified from Li et al. (2018). Habit was derived from three items from Venkatesh et al. (2012). Service rule commitment was assessed by five measures taken from Wang et al. (2011). Request behavior was evaluated according to four items abstracted from previous studies (Teng et al., 2020) with several words. The constructs were gauged with a seven-point Likert scale, in which 1 indicates “strongly disagree” and 7, “strongly agree.” Table 2 shows these items in detail.

**Table 2:** Factor items and loadings.

Construct	Item	Mean	S. D.	Factor loading^a^	Critical ratio
Normative legitimacy	1. I think the request of sharing tables is acceptable.	4.284	2.009	0.769	25.91
	2. I think the request of sharing tables is reasonable.	4.398	2.041	0.769	26.31
	3. I think the request of sharing tables is justified.	4.299	2.008	0.864	35.38
Role confidence	1. I am confident to provide service.	4.410	1.969	0.761	26.76
	2. I feel content with my manner in which I provide service.	4.463	2.007	0.824	34.58
	3. I can give the best service.	4.457	1.905	0.742	24.77
	4. I know all about how to serve customers.	4.336	1.899	0.717	22.61
	5. I can deal with customers’ difficult behaviors well.	4.377	2.012	0.666	18.86
Expected responsiveness	1. I know regarding the timing of service delivery.	4.380	1.908	0.750	24.09
	2. I provide customers with immediate service.	4.352	1.973	0.731	22.70
	3. I consistently offer my assistance to customers.	4.395	1.935	0.769	25.79
	4. I always make time to deal with customer requests.	4.457	1.918	0.760	25.11
Expected technical quality	1. I want to complete the service successfully.	4.448	1.975	0.703	19.57
	2. There should be no disturbance during my service.	4.488	1.907	0.763	23.09
	3. The service will be finished without interruption.	4.364	1.917	0.827	27.42
Habit	1. Making the request to share tables has become a habit for me.	4.414	1.798	0.940	64.78
	2. I am addicted to requesting customers to share tables.	4.617	1.767	0.866	47.39
	3. Requesting customers to share tables has become natural to me.	4.426	1.851	0.822	38.10
Service rule commitment	1. While interacting with customers, adhering strictly to these service rules can be challenging. (R)	3.762	1.942	0.753	27.68
	2. To be perfectly candid, whether I follow these service rules or not is of little concern to me. (R)	3.457	1.918	0.802	34.49
	3. While attending to customers, I ensure adherence to my company’s rules for customer service.	3.580	2.002	0.889	54.35
	4. While assisting customers, it would not require much for me to disregard service rules. (R)	3.645	1.977	0.770	29.67
	5. I believe it is beneficial to adhere to the service rules provided by my organization.	3.620	2.057	0.713	23.50
Request behavior	1. I often request customers to share tables.	4.315	1.943	0.785	32.22
	2. I frequently ask customers to share tables.	4.355	1.971	0.939	68.73
	3. I like to ask customers to share tables.	4.370	1.868	0.777	31.49
	4. It is a good choice to ask customers to share tables.	4.336	1.857	0.717	24.21

^a^All loadings are significant at the 0.001 level.

^b^χ2 (358.091)/df (303) = 1.182, *p* < 0.05, CFI = 0.988, TLI = 0.986, SRMR = 0.036, and RMSEA = 0.024.

Considering the original items are in English, three Ph.D. students and two marketing professors adapted the items and checked them one-by-one to ensure content validity. Then, to keep the meaning consistent, we conducted back-translations, starting with converting from English into Chinese, and then reverting from Chinese into English. The measures of the Chinese version were also assessed by seven FLEs and two marketing professors to ensure relevance for this research context.

## 5 Results

### 5.1 Measurement model

In MPLUS 7.0, we employed confirmatory factor analysis (CFA) to examine the measurement attributes of the constructs in question. The CFA yielded strong fit indices for the data with χ2 (358.091)/df (303) = 1.182, *p* < 0.05, comparative fit index (CFI) = 0.988, Tucker-Lewis index (TLI) = 0.986, standardized root mean squared residual (SRMR) = 0.036, and root mean square error of approximation (RMSEA) = 0.024. With all items featuring standardized loadings above 0.6, the respective composite reliability (CR) and Cronbach’s alpha values for each construct are above the 0.8 mark (see Tables 2, 3), thus supporting each construct’s adequate reliability and convergent validity (Hair Jr et al., 2010). Meanwhile, construct interrelations are below the square roots of the average variance extracted (AVE), with all AVE values exceeding 0.5 (see Table 3), thus indicating an adequate convergent validity.

**Table 3:** Reliability and discriminant validity.

Construct	CR	Cronbach’s alpha	AVE	1	2	3	4	5	6	7
1. Normative legitimacy	0.844	0.839	0.643	**0.802**						
2. Role confidence	0.860	0.859	0.553	0.300	**0.744**					
3. Expected responsiveness	0.839	0.839	0.567	0.343	0.410	**0.753**				
4. Expected technical quality	0.809	0.806	0.587	0.378	0.274	0.332	**0.766**			
5. Habit	0.909	0.907	0.770	0.224	0.257	0.176	0.240	**0.877**		
6. Service rule commitment	0.890	0.887	0.620	−0.499	−0.586	−0.558	−0.413	−0.316	**0.788**	
7. Request behavior	0.882	0.875	0.654	0.519	0.528	0.485	0.485	0.234	0.613	**0.809**

The values below the diagonal are correlation coefficients, all of which are significant at the 0.010 level, while the bolded numbers on the diagonal indicate the square roots of the average variance extracted (AVE).

### 5.2 Structural model results

**Figure 2:**
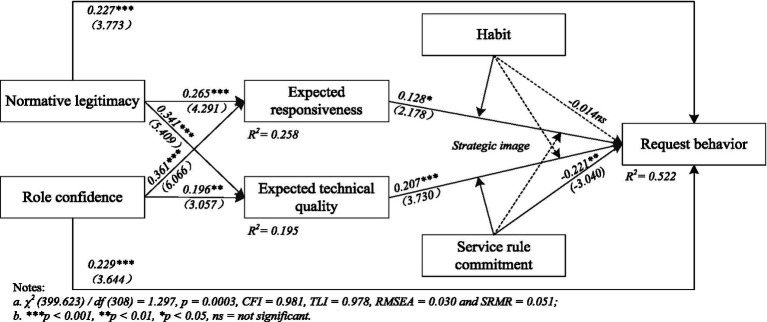
Results of the structural model.

We utilized MPLUS 7.0 to develop a structural model and assess the theoretical linkages among the constructs. As Figure 2 shows, the structural model yielded adequate fit indexes with χ2 (399.623)/df (308) = 1.297, *p* = 0.0003, CFI = 0.981, TLI = 0.978, RMSEA = 0.030, and SRMR = 0.051. The standardized path coefficients and associated T-values indicate that normative legitimacy (*β* = 0.277, *p* < 0.001), role confidence (*β* = 0.229, *p* < 0.001), expected responsiveness (*β* = 0.128, *p* < 0.05), and expected technical quality (*β* = 0.207, *p* < 0.001) play a key role in fostering positive developments in request behavior, while service rule commitment (*β* = −0.221, *p* < 0.01) contributes to a substantial adverse consequence for the request behavior, confirming the predictions of hypotheses H1 to H4 and H6 in turn. Conversely, the link between habit and request behavior (*β* = −0.014, *p* > 0.10) proves to be insignificant, thereby not validating H5. Meanwhile, both normative legitimacy (*β* = 0.265, *p* < 0.001; *β* = 0.341, *p* < 0.001) and role confidence (*β* = 0.361, *p* < 0.001; *β* = 0.196, *p* < 0.01) favorably contribute to expected responsiveness and expected technical quality. Thus, H7a to H7d are supported.

### 5.3 Mediation examination

Adhering to the methodologies proposed by Zhao et al. (2010) and Zhang et al. (2020), we built three structural models to conduct mediation tests in MPLUS 7.0 (see Table 4). As Model 1 shows, the relationships between independent variables (i.e., normative legitimacy and role confidence) and the dependent variable (i.e., request behavior) are significant (*β* = 0.406, *p* < 0.001; *β* = 0.389, *p* < 0.001). Model 2 indicates that the relationships between the dependent and mediating variables (i.e., expected responsiveness and expected technical quality) are both significant (*β* = 0.400, *p* < 0.001; *β* = 0.395, *p* < 0.001), meanwhile the relationships between the independent and mediating variables are all significant (*β* = 0.364, *p* < 0.001; *β* = 0.263, *p* < 0.001; *β* = 0.209, *p* < 0.01; *β* = 0.347, *p* < 0.001). Lastly, we added independent variables and mediating variables equally in Model 3, which shows that the coefficient values between the independent variables (normative legitimacy and role confidence) and the dependent variable (request behavior) decrease. Nonetheless, the results are apparent when introducing mediating variables (expected responsiveness and expected technical quality). As demonstrated by the principle formulated by Baron and Kenny (1986), we find that expected responsiveness and expected technical quality partially (trajectory image) mediate the impacts of normative legitimacy and role confidence (value image) on request behavior.

**Table 4:** Results of the mediating effects.

Construct	Model 1	Model 2			Model 3
	IV → DV	IV → MV	IV → MV	MV → DV	IV + MV → DV
Role confidence	0.406***	0.364***	0.209**		0.305***
Normative legitimacy	0.389***	0.263***	0.347***		0.272***
Expected responsiveness (ER)				0.400***	0.188**
Expected technical quality (ETQ)				0.395***	0.235***
R^2^	0.410	0.258	0.207	0.386	0.505
χ^2^	73.418	MV = ER	MV = ETQ	228.325	166.734
df	51			145	142
χ^2^/*df*	1.440			1.575	1.174
p	0.0216			0.0000	0.0765
CFI	0.989			0.971	0.992
TLI	0.985			0.966	0.990
RMSEA	0.037			0.042	0.023
SRMR	0.038			0.071	0.034

DV: dependent variable; IV: independent variable; MV: mediating variable; **p < 0.01,***p < 0.001.

### 5.4 Moderation analysis

**Figure 3:**
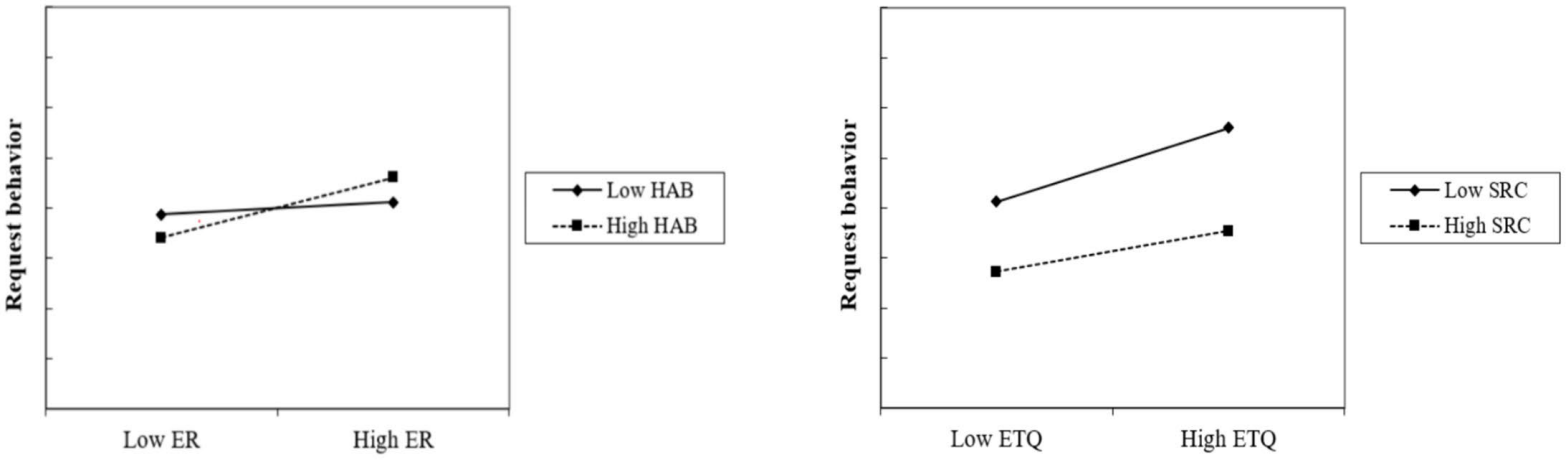
Results of moderation examinations.

The XWITH command in MPLUS 7.0 was used to generate the new interaction terms to verify moderation effects, following the method of Li et al. (2020). As Table 5 shows, we built Model 2, Model 3, Model 4, and Model 5 to add the four interaction terms. The outcomes indicate that the interaction terms of habit and expected responsiveness (*γ* = 0.119, *p* < 0.001) and of service rule commitment and expected technical quality (γ = −0.084, *p* < 0.05) are significant, while the interaction terms of habit and expected technical quality (γ = 0.042, *p* > 0.10) and of service rule commitment and expected responsiveness (γ = −0.018, *p* > 0.10) are not significant. Consequently, the support for hypotheses H8a and H8d is established, but H8b and H8c are not supported. Habit moderating the influence relationship between expected responsiveness and request behavior is shown in the left-hand graph in Figure 3. Service rule commitment moderating the influence relationship between expected technical quality and request behavior is shown in the right-hand graph in Figure 3.

**Table 5:** Results of the moderation effects.

Construct	Model 1	Model 2	Model 3	Model 4	Model 5
Expected responsiveness (ER)	0.171**	0.180 **	0.179**	0.184**	0.186**
Expected technical quality (ETQ)	0.254***	0.272***	0.282***	0.280***	0.287***
Habit (HAB)	0.013 ns	0.005 ns	0.013 ns	0.011 ns	0.005 ns
Service rule commitment (SRC)	−0.406***	−0.439 ***	−0.420**	−0.426***	−0.432***
HAB × ER		0.119***			
HAB × ETQ			0.042 ns		
SRC × ER				−0.018 ns	
SRC × ETQ					−0.084*

The fit indexes for Model 1 are χ2 (189.617)/df (142) = 1.335, *p* < 0.01, CFI = 0.986, TLI = 0.983, SRMR = 0.035, and RMSEA = 0.032; Model 2, Model 3, Model 4 and Model 5 specify ALGORITHM = INTEGRATION for moderation examinations with MPLUS 7.0; **p < 0.01; ***p < 0.001; ns: not significant.

## 6 Discussion

### 6.1 Key findings

Our study explores the factors that influence employees’ fuzzy request behavior. Based on the image theory, we put forward a model that explains the impetus behind the initiation of this request behavior.

First, this request behavior is driven by normative legitimacy, role confidence, expected responsiveness, expected technical quality, and service rule commitment, which were constructed from the image theory. The findings show that value image (i.e., normative legitimacy and role confidence) and trajectory image (i.e., expected responsiveness and expected technical quality) have positive influences on request behavior. The results confirm some driving effects of these factors on both behavior and response, which are proposed by Wang et al. (2012). In contrast, service rule commitment exerts an inverse impact on employees’ request behavior, that is, the more the employees pay attention to service scripts, the more they will serve customers according to the rules and the less likely they are to make fuzzy requests. However, the effect of habit was not significant, which differs from previous findings (Li et al., 2018). The possible reason is that the role of habit is uncertain in the case of dynamic interaction fluctuations. While habit is the automatic behavior pattern that employees gradually form when they handle routine and repetitive tasks in the long-term work process (Li et al., 2018), it cannot function when FLEs are handling a specific and sudden interaction, especially to better complete the service. In other words, FLEs want instant responses rather than relying on inherent habits to address a new circumstance or service fluctuation when they deliver fuzzy requests (Li et al., 2021).

Second, the study identifies that trajectory image mediates the effect of value image on request behavior. As shown in the results, normative legitimacy of the fuzzy request and the role confidence of FLEs influence request behavior through expectations (i.e., expected responsiveness and expected technical quality) in the service interaction. In the service interaction, FLEs’ confidence and the request behavior’s legitimacy will make employees more proactive and responsive toward customers (Nguyen et al., 2023; Wang et al., 2012), which causes employees to make requests of customers during service provision in order to achieve the desired service quality and improve customer satisfaction. This finding also validates some findings proposed by Li et al. (2018). The findings indicate that FLEs’ hope (trajectory image) for service quality expectations is a vital path mechanism for the influence of service principles (value image) on FLEs’ behavior. It extends the understanding of the mediation relationship between value image and trajectory image (Beach, 1993b) of explaining FLEs’ request behavior.

Third, this study identifies separate moderating effects of habit and service rule commitment. The particularly interesting moderating effects show that habit just moderates process quality expectations while service rule commitment just moderates outcome quality expectations. Specifically, FLEs’ habit links the strong relationship between quick service expectation and request behavior, as the behavior is seen as a solution of quick response (Sun et al., 2017). When FLEs’ habit is strong, their expectations for quick service will increase their request behavior tendency. However, FLEs’ habit has no significant moderating effect on the relationship between service outcome expectations and request behavior. This indicates that when FLEs exhibit request behavior, their core purpose is still to receive a quick response during the service process, rather than ignoring service quality (Oliveira, 2024). In other words, they are aware that this habit-based reaction has an (unexpected) impact on expected technical quality. This means habit/past experience may not provide a clear guide to the construct service standard, since fuzzy requests often lack clear patterns or precedents. Meanwhile, service rule commitment has no (a) significant moderating effect on the relationship between expected responsiveness (technical quality) and request behavior. The findings indicate that service rule commitment influences outcomes rather than processes’ expectation effects. It means FLEs empathize with service outcome linking their request behavior, as they need to focus on the normativity and consistency of employee behavior, which is regulated by service rule (Lajante et al., 2023). However, it is common that service script generally describes how to respond to customers and FLEs see fuzzy requests as an urgent response rather than a rule-based solution, which has no moderating effect on expected responsiveness. These findings indicate that FLEs’ characteristics and service provision have different impact paths on the relationships between FLEs’ expectations and request behavior. It also reveals that strategic image from different objects (i.e., habit and service rule commitment) guide FLEs in achieving service success in different styles.

### 6.2 Theoretical contributions

Our scholarly work delivers a set of theoretical insights. Notably, this research is one of the first that focuses on requests from FLEs’ perspective and is the first to identify the determinants of request behavior. Specifically, different from Wang et al. (2012), Beatty et al. (2016), and Li et al. (2022), who explore fuzzy requests from a customer’s perspective, this study shifts the focus to the employees’ perspective to explore how FLEs’ request behaviors emerge. It is also different from some studies focused on FLEs’ requests (e.g., Li et al., 2018; Teng et al., 2020) through identifying the determinants of FLEs’ request behaviors and driving mechanism and thus deepens our understanding of the emergence of fuzzy requests from a new angle. It provides empirical evidence of new origin of service fluctuation and calls for more attention to recognize the service fluctuation, which can be generated by FLEs and extends the guide for managing service interaction from two sides.

Second, we put forward a model to explain the mechanism driving employees’ request behavior. Different from previous studies, which mostly see FLEs’ behavior as a rational decision result (Ganuthula, 2024; Li et al., 2021) or examine requests from the customers’ perspective (Li et al., 2022), this study analyzes FLEs’ request behavior from a perspective of the interaction relationship and content (Morrell, 2004). Through hypotheses, mediation, and moderation examinations, the study provides a clear theoretical perspective that compensates for the current fragmentation of the understanding of request behavior. Specifically, we identify the mediation effects of expectations of service quality on the service provision (such as normative legitimacy or role confidence) on behavior decision, which have been ignored in explanations for fuzzy requests. Meanwhile, it is the first to identify separate moderating effects of habit and service rule commitment on expectations and behavior. These results provide a more comprehensive understanding of the complex dynamics that drive request behavior in service fluctuation settings.

Third, this study extends the application of image theory beyond its traditional use in decision-making research (Kuehn, 2016; Nelson, 2004; Pesta et al., 2005). Different from previous studies where image theory primarily focused on analyzing individual decisions within regular or traditional service contexts (Beach and Mitchell, 1987), we adopt it to explore employees’ behavior during service fluctuation contexts. We abstracted six variables aligned with the three images of image theory and found that trajectory image has a mediating role that influences FLEs’ request behavior, and relationships outlined by strategic theory (i.e., habit and service rule commitment) have moderating effects on trajectory image influencing behavior. These results highlight the unique role of image theory in understanding service dynamics and contribute to the broader theoretical framework by revealing new internal relationships within the theory and their application to employees’ behavior.

### 6.3 Practical implications

This study offers numerous practical implications. First, organizations should recognize the phenomenon of FLEs’ fuzzy request (behavior) and adopt such requests as a potential tool to improve service performance. Organizations can check the drivers of FLEs’ request behavior using our proposed model and train and guide them to promptly take advantage of requests in some service context, as these requests are unavoidable and FLEs’ request behavior can improve service efficiency or extend the method to achieve service goals (Li et al., 2018; Teng et al., 2020). In addition, organizations should be aware of the characteristics of fuzzy requests and be mindful of circumstances about how FLEs can avoid service failures throughout the process of service provision (Kostopoulos et al., 2014; Li et al., 2018). In other words, the success and long-term survival of a service organization depend on the behavior of its employees (Wang et al., 2024). Organizations should balance the rule and flexibility to provide employees with the flexibility and support to respond to unexpected situations. A feedback mechanism can be used to regularly evaluate the effectiveness of service provisions and rules, thus increasing successful cases of request behavior. The study can also help service providers to correctly understand, comprehend, and put forward fuzzy requests, and subsequently enhance service quality to attain customer satisfaction in a specific reference direction.

Second, the proposed factors and their effects contribute to FLEs making decisions for the request behavior. Specifically, the study identifies some positive factors (normative legitimacy, role confidence, expected responsiveness, and expected technical quality) and negative factors (service rule commitment) for FLEs’ request behavior. Thus, FLEs should comprehensively consider the aspects mentioned above regarding whether they can exhibit request behavior. Meanwhile, FLEs can enhance their role confidence through learning from service interactions and learn service scripts and organizational specifications in depth to understand values and norms for evaluating the legitimacy of their request behavior. In addition, FLEs can establish a better relationship with customers by further communicating with them and focusing on their needs in daily work to improve expectations of the responsiveness in process and service quality. In addition, FLEs should be aware that relying solely on habits can lead to inappropriate decisions. Especially in the face of uncertain situations, employees should avoid making decisions solely based on past experience and habits but should consider new information and needs and adopt more flexible coping strategies. Furthermore, FLEs should have a clear understanding of service rules and find a balance between rules and flexibility to respond to emergencies. It can not only guide them in service interaction but also help them make prompt decisions in service fluctuations to improve service quality. In summary, FLEs should continue to learn and improve in the service process, fully enhance the impact of role confidence, understand service norms and rules, set expectation responsiveness, maintain strategic thinking, and so on, to make more efficient and appropriate decisions, thus improving the overall service level.

Third, the emergence of FLEs’ request behavior would lead to public perceptions of service fairness and decrease customer trust. Organizations should consider the social impact of fuzzy requests and service reputations. If customers encounter opaque or unreasonably fuzzy requests or frequently receive such requests, this may reduce their trust and overall satisfaction and make them doubt the organization’s promises. Considering that these requests usually occur with uncertainty (Li et al., 2022) and are an unavoidable phenomenon in terms of service fluctuation, both organizations and FLEs should balance rule commitment and handling flexibility to meet expectations. Meanwhile, organizations should take measures to learn from such requests, and see them as a source of service innovation (Li et al., 2022) rather than the means of taking advantaging of customers. Some service policies can be modified based on these requests to assure customer trust and avoid feelings of unfair treatment. In addition, some industrial policies (mainly reflected in the aspects of service transparency, industry norms, and consumer rights protection) can be timely made to guide service providers to manage request behaviors. Thus, service quality can be improved while service providers increase service efficiency and reduce the wastage of service resources.

Overall, the study provides critical implications for organizations seeking to manage fuzzy request behaviors while maintaining compliance with existing regulations and internal guidelines. First, organizations can embed controlled discretion into their standard operating procedures by clearly defining the boundary conditions under which frontline employees are authorized to initiate fuzzy requests. This approach allows flexibility in service delivery without undermining rule consistency. Second, managers can develop decision-support frameworks that help employees assess the legitimacy, risk, and appropriateness of request behaviors in real time, thereby aligning discretionary actions with organizational values and industry regulations. Third, organizations should integrate these findings into training and development programs by equipping employees with both technical skills and ethical judgment capabilities, ensuring that adaptive behaviors are exercised within legal and institutional constraints. Finally, firms can establish monitoring and feedback mechanisms to continuously evaluate whether fuzzy request behaviors remain aligned with regulatory requirements and service standards. Through these mechanisms, organizations can achieve a balance between flexibility and compliance, thereby improving service responsiveness without increasing organizational or legal risk.

### 6.4 Limitations and future research directions

While the study can yield many valuable findings and implications, it also has some limitations that might prompt further research. One limitation is the lack of generalizability as this study has a low level of external validity due to the sample composition. While focusing solely on Chinese chain restaurants, further investigation could be extended to chain restaurants in other countries or regions, or comparative analyses could be conducted among different types of catering enterprises. Additionally, the research could further explore the influence of culture, policies, and market environment on the research conclusions, in order to gain a more comprehensive understanding of the relevant phenomena. Second, the data this study uses are relative in cross-sections, and samples were gathered during a designated timeframe, so it is not known whether there are dynamic changes in the process. Further research can use longitudinal study designs or panel data designs to better understand FLEs’ behavior and changes. Moreover, future studies could employ multi-source data, experimental designs, and field interventions to further validate and refine the proposed model. Finally, our study ignored some associated potential moderating variables, including contextual (e.g., specific organizational culture and environment) and individual (e.g., personal traits, emotions) factors, which can be examined in future research.

## Data Availability

The raw data supporting the conclusions of this article will be made available by the authors, without undue reservation.
